# Reducing farnesyl diphosphate synthase levels activates Vγ9Vδ2 T cells and improves tumor suppression in murine xenograft cancer models

**DOI:** 10.3389/fimmu.2022.1012051

**Published:** 2022-10-05

**Authors:** Mei-Ling Liou, Tyler Lahusen, Haishan Li, Lingzhi Xiao, C. David Pauza

**Affiliations:** ^1^ American Gene Technologies International Inc., Rockville, MD, United States; ^2^ Viriom Inc., Rockville, MD, United States

**Keywords:** T-cell, gamma delta T-cell, cancer, tumor, immunotherapy, shRNA, FDPS

## Abstract

Human Vγ9Vδ2 T cells are attractive candidates for cancer immunotherapy due to their potent capacity for tumor recognition and cytolysis of many tumor cell types. However, efforts to deploy clinical strategies for Vγ9Vδ2 T cell cancer therapy are hampered by insufficient potency. We are pursuing an alternate strategy of modifying tumors to increase the capacity for Vγ9Vδ2 T cell activation, as a means for strengthening the anti-tumor response by resident or ex vivo manufactured Vγ9Vδ2 T cells. Vγ9Vδ2 T cells are activated *in vitro* by non-peptidic antigens including isopentenyl pyrophosphate (IPP), a substrate of farnesyl diphosphate synthase (FDPS) in the pathway for biosynthesis of isoprenoids. In an effort to improve *in vivo* potency of Vγ9Vδ2 T cells, we reduced FDPS expression in tumor cells using a lentivirus vector encoding a short-hairpin RNA that targets FDPS mRNA (LV-shFDPS). Prostate (PC3) or hepatocellular carcinoma (Huh-7) cells transduced with LV-shFDPS induced Vγ9Vδ2 T cell stimulation *in vitro*, resulting in increased cytokine expression and tumor cell cytotoxicity. Immune deficient mice implanted with LV-shFDPS transduced tumor cells showed dramatic responses to intraperitoneal injection of Vγ9Vδ2 T cells with strong suppression of tumor growth. *In vivo* potency was increased by transducing tumor cells with a vector expressing both shFDPS and human IL-2. Tumor suppression by Vγ9Vδ2 T cells was dose-dependent with greater effects observed in mice injected with 100% LV-shFDPS transduced cells compared to mice injected with a mixture of 50% LV-shFDPS transduced cells and 50% control (no vector) tumor cells. Delivery of LV-shFDPS by intratumoral injection was insufficient to knockdown FDPS in the majority of tumor cells, resulting in insignificant tumor suppression by Vγ9Vδ2 T cells. Thus, Vγ9Vδ2 T cells efficiently targeted and suppressed tumors expressing shFDPS in mouse xenotransplant models. This proof-of-concept study demonstrates the potential for suppression of genetically modified tumors by human Vγ9Vδ2 T cells and indicates that co-expression of cytokines may boost the anti-tumor effect.

## Introduction

Human Vγ9Vδ2 (hereinafter referred to as Vδ2) T cells are a unique population in peripheral blood with frequencies ranging from 0.5-10% of peripheral blood mononuclear cells (PBMC). Due to unique features of their T cell receptor, these are often considered to have features representing both innate and adaptive immunity (1). The T cell receptor (TCR) on Vδ2 T cells is composed of V gamma 9), a specific J segment (JP) and the V delta 2 chains capable of responding to non-peptide antigens independently of antigen presentation by major histocompatibility complex proteins (MHC) (2, 3). Non-peptide antigens capable of stimulating Vδ2 T cells are known as phosphoantigens (PAgs) include (E)-4-hydroxy-3-methyl-but-2-enyl pyrophosphate (HMBPP) found in bacterial, fungal and protozoan pathogens, and isopentenyl pyrophosphate (IPP), an intermediate metabolite in the mevalonate pathway for biosynthesis of isoprenoids (**Supplementary Figure 1**) (4–7).

PAg recognition depends on cell surface molecules from the butyrophilin family, most importantly 3A1 and 3A2 (8, 9). In our current understanding, butyrophilin binds PAg *via* a cytoplasmic domain, resulting in a conformational change to the ectodomain that is recognized by Vδ2 T cells (10–12). Considering that butyrophilins are widely expressed and tumor cells upregulate the mevalonate pathway to meet an increasing need for membrane synthesis (13, 14), Vδ2 T cells respond to many types of malignant cells and may participate in tumor immunosurveillance through their recognition of PAg (15–17). The common expression of butyrophilins and upregulation of the mevalonate/cholesterol pathway allows for broad recognition of tumors by Vδ2 T cells (18–20). Accordingly, Vδ2 T cells are increasingly appreciated as a key component in natural tumor immunity and a potential target for cell-based therapy against cancers due to their ability to recognize malignant cells, infiltrate tumors, and release cytotoxic and pro-inflammatory cytokines (21–24). Both adoptive transfer and *in vivo* activation of Vδ2 T cells have been safe during clinical trials making them a promising cell therapy against various tumors such as lymphoma, myeloma (25), hepatocellular, and colorectal carcinoma (26), and prostate (23), lung (27), colon (28), breast (24), and ovary cancers (29).

Vδ2 T lymphocytes can be activated by PAg producing accessory cells, such as immune presenting cells and tumor cells, or self-activate through exogenous PAgs (30). The activated Vδ2 T cells are cytolytic against tumor cells, secrete the inflammatory cytokines TNFα and IFNγ among others, release cytolytic proteins perforin and granzyme B, and are effector cells for antibody-dependent cell mediated cytotoxicity (ADCC) (31).

Nitrogen-containing-bisphosphonates (NBPs) have immunomodulatory properties including activation of Vδ2 T cells. NBPs are inhibitors of farnesyl diphosphate synthase (FDPS), an enzyme in the mevalonate pathway (**Supplementary Figure 1**). NBPs compete for IPP binding on FDPS leading to competitive enzyme inhibition, IPP accumulation and Vδ2 T cell activation (32–34). NBPs are used clinically to treat osteoporosis and osteolytic bone lesions. Pharmaceutical NBPs, such as zoledronic acid (Zomata), pamidronate (Aredia) and Alendronic acid (Fosamax) are used to treat osteoporosis and are reported to have anti-tumor effects in metastatic bone cancer patients (35–37). Given the anti-tumor effect exhibited by NBPs, in addition to the fact that some tumors overproduce IPP, *in vitro* data showed that treatment of tumor cells with NBPs leads to activation of Vδ2 T cells and increased cytolytic responses against tumor cells (38).

Existing approaches for exploiting Vδ2 T cells in cancer therapy utilize soluble activators of Vδ2 T cells such as NBPs or PAg to increase the potency of Vδ2 T cells against tumor cell targets (39). Another approach to activating Vδ2 T cells uses RNA interference with a small hairpin RNA (shRNA) stem-loop structure to knockdown FDPS mRNA in tumor cells, thus causing IPP accumulation and Vδ2 T cell activation (6, 40, 41). As a proof of concept, we delivered shFDPS to cancer cells using a lentivirus vector. The large carrying capacity of lentiviral vectors also allowed us to co-express shFDPS and immune-stimulating factors on individual lentivirus vector constructs. Several cytokines are known to enhance Vδ2 T cell activation or increase Vδ2 T cell expansion. IL2 and IL15 increase the activity of Vδ2 T cells in response to PAg or NBPs (42, 43) and IL2 increases the expansion of Vδ2 T cells in response to zoledronic acid (44, 45). Expressing IL2 in addition to FDPS shRNA in a lentiviral vector might enhance the potency of Vδ2 T cells against cancer (24).

The combination of NBPs and Vδ2 T cell treatments used in xenograft mouse models were tested in several human tumor types including melanoma (46), glioma (47), neuroblastoma (48), pancreatic (46, 49), and prostate cancer (49). However, there are two limitations to using NBPs for activating Vδ2 T cells *in vivo*. One is that soluble NBPs are absorbed by binding to hydroxyapatite on the bone surface, thus reducing bioavailability, and the second is that NBPs cannot be targeted to the tumors. Consequently, we constructed a lentiviral vector expressing shFDPS (LV-shFDPS) to determine if reducing FDPS mRNA and protein expression in prostate and hepatocellular tumors ex vivo or *in vivo* would lead to tumor suppression by Vδ2 T cells from healthy human donors. We also tested whether FDPS knockdown in addition to NBP treatment could increase the activation and cytolytic activity of Vδ2 T cells and whether this would be enhanced further with concurrent tumor cell expression of IL2.

## Materials and methods

### Isolation and cryopreservation of human PBMC

Leukopaks from anonymous donors were obtained from the New York Blood Center (New York, NY, USA). PBMC were isolated using density gradient centrifugation using a Ficoll-Paque Plus gradient (Sigma-Aldrich, St. Louis, MO, USA). The PBMC were transferred to a new tube and washed twice with PBS. The resulting cell pellet was resuspended in culture medium composed of RPMI 1640 complete medium: RPMI 1640 medium (Thermo Fisher Scientific, Burlington, MA, USA), 10% Fetal Bovine Serum (FBS) (Gemini, Sacramento, CA, USA), and 10,000 U/mL Penicillin-Streptomycin (Thermo Fisher Scientific). PBMC were cryopreserved in 90% FBS + 10% DMS0 (Sigma-Aldrich) at a final concentration of 3 X 10^7^ cells/mL. The PBMC were placed at -80°C for overnight and then transferred to liquid nitrogen for long term storage.

### Expansion of Vδ2 T cells from cryopreserved human PBMC

3 X 10^7^ cryopreserved PBMC were thawed in a 37°C water bath and washed with RPMI 1640 medium. The PBMC were resuspended at a concentration of 1 X 10^7^cells/mL in 10 mL RPMI 1640 medium supplemented with 1 μM zoledronic acid (ZA) (Sigma-Aldrich) and 0.1 mg/mL recombinant human IL2 (Thermo Fisher Scientific). The cells were expanded in T25 or T75 tissue culture flasks (Greiner Bio, VWR, Radnor, PA, USA) for a minimum of two weeks prior to use for *in vitro* and *in vivo* experiments. During the first 10 days of expansion, the medium was supplemented with 0.1 mg/mL of recombinant human IL2 twice in the first week after which IL2 supplementation was continued at 0.01 mg/mL. After a minimum of two weeks in culture, the mixed cell population contained 20-90% Vδ2 T cells as determined by flow cytometry.

### Cultivation of cancer cell lines

PC3 prostate carcinoma cells were obtained from the American Type Culture Collection (ATCC, Manassas, VA, USA) and Huh-7 liver hepatocellular carcinoma cells were obtained from the Japanese Collection of Research Bioresources (JCRB Cell Bank, Osaka, Japan). The cells were thawed and passaged in DMEM complete medium: DMEM (Thermo Fisher Scientific), 10% FBS (Gemini), and 10,000 U/mL Penicillin-Streptomycin (Thermo Fisher Scientific). Cells were seeded into T-75 flasks (Greiner Bio, VWR) and cultured in a 37°C incubator with 5% CO_2_.

### Transduction of cancer cells and coculture with Vδ2 T cells

PC3 or Huh-7 cells were seeded at 0.5 X 10^6^ cells/well in a 6-well plate followed by incubation overnight. The next day, when the cells had reached around 50% confluency, the cells were transduced with LV control, LV-shFDPS or LV-shFDPS-IL2 using a multiplicity of infection (MOI) of 5 or 10. Three days after transduction, the transduced PC3 or Huh-7 cells were collected, centrifuged, and resuspended in fresh, complete DMEM medium. The PC3 or Huh-7 cells were added at 5 X 10^5^ cells/100 µL in wells of 96-well U-bottom plates. Next, 1 X 10^6^/100 µL Vδ2 T cells were added to the wells and cytokine secretion was arrested by adding the GolgiPlug reagent per the manufacturer’s instructions (BD Biosciences, Franklin Lakes, NJ, USA). The coculture was incubated in a 37°C cell incubator for 4 h, whereupon cells were collected and cytokine expression was measured by flow cytometry.

### Measuring FDPS expression by immunoblotting

293T cells were seeded at 8 X 10^5^ cells in each well of a 6-well plate and cultured in 1.5 mL of complete DMEM medium in a 37°C incubator at 5% CO_2_ overnight. The next day, lentivirus vector was added to the cells at a MOI of 2.5, 5, 10 or 20 for 48 h. For PC3 cells, in addition to lentivirus, polybrene (Sigma-Aldrich) was added to the cell medium at a concentration of 2 μg/mL. Cells were lysed in 1% NP-40 lysis buffer containing a Pierce Protease Inhibitor Tablet (Thermo Fisher Scientific). Protein lysates were prepared in 1x NuPAGE LDS sample buffer (Thermo Fisher Scientific), samples were heated for 10 min at 70°C, and proteins were separated with 4-12% NuPAGE Bis-Tris gels (Thermo Fisher Scientific). Proteins were transferred to polyvinylidene fluoride (PVDF) membranes (MilliporeSigma) and probed with the antibodies anti-FDPS (Bethyl Laboratories Fortis Life Sciences, Waltham, MA, USA) and β-actin (MilliporeSigma). Anti-mouse or rabbit secondary antibody conjugated with horseradish peroxidase (HRP) (Bio-Rad, Hercules, CA, USA) was visualized with the Immobilon Western HRP substrate (MilliporeSigma) and detected with the LI-COR C-DiGit Blot Scanner (Lincoln, NE, USA).

### Measurement of IL2 by ELISA

PC3 cells were transduced with lentivirus at 5 MOI in addition to 2 μg/mL of polybrene. The cell medium was changed after 6 h and then collected after 3 days whereupon IL2 expression was determined with a human IL2 ELISA kit (Thermo Fisher Scientific).

### Reverse transcriptase (RT) PCR and real-time qPCR

Cells were collected and RNA was extracted with the RNeasy kit (Qiagen, Germantown, MD, USA). The reverse transcription reaction was done using 1.4 μg of RNA and the Vilo SuperScript cDNA synthesis kit (Thermo Fisher Scientific) on a Veriti 96-well thermal cycler (Thermo Fisher Scientific). The PCR steps were according to the following: 25°C for 10 min, 42°C for 60 min, and 85°C for 5 min. For the Real-Time qPCR assays, 1.5 μL of the RT-PCR reaction was mixed with 1 μL of FDPS (Fwd: 5”-GTGCTGACTGAGGATGAGATG-3’, Rev: 5”-CCGGTTATACTTGCCTCCAAT-3”, Fam probe: 5’-TAGCTCTCCTATCTCTGGGTGCCC-3’) and actin (Fwd: 5’-GGACCTGACTGACTACCTCAT-3”, Rev: 5’-CGTAGCACAGCTTCTCCTTAAT-3”, Yakima probe: 5’-AGCGGGAAATCGTGCGTGAC-3’) primers and probes at 0.5 μM and the reaction components of the TaqMan Fast Advanced Master Mix (Thermo Fisher Scientific). The qPCR reactions were performed on a QuantStudio3 Real-Time PCR system (Thermo Fisher Scientific) according to the following steps: 50°C for 2 min, 95°C for 20 secs, 40 cycles of 95°C for 1 sec and 60°C for 20 secs. Data were collected and analyzed with QuantStudio Design and Analysis software (Thermo Fisher Scientific).

### Flow cytometry analysis

The 96-well U-bottom plate containing the co-cultured cells was centrifuged, followed by removal of the supernatant and resuspension in 50 µL of flow staining buffer (1X PBS magnesium/calcium depleted + 1% FBS) containing PE-labeled anti-human Vδ2 antibody (BioLegend, San Diego, CA, USA). Next, the cells were treated with 50 µL of fixation buffer (BD Biosciences) before washing with 1X perm/wash solution (BD Biosciences). Then cells were resuspended in 50 μL of APC-labeled anti-human TNFα or IFNγ (BioLegend). The cells were incubated at 4°C for 20 min in the dark and washed 2 times with 250 μL of 1X perm/wash solution. Flow cytometry data were acquired on a FACS Calibur (BD Biosciences) and analyzed using FlowJo software.

### Labeling of cancer cells with calcein AM

PC3 or Huh-7 cells were seeded in 6-well plates at 4 X 10^5^ cells per well and incubated overnight. The next day, the cells were transduced with lentiviral vector stocks at a MOI of 5 or 10. For PC3 cells, in addition to lentivirus, polybrene was added to the cell medium at a concentration of 2 μg/mL. After 48 h, the medium was changed with or without 1 μM ZA for overnight incubation. The next day, 5 μM of Calcein AM (Thermo Fisher Scientifc) were added to the medium and cells were placed in a 37°C cell incubator for 20 min. Next, the cells were washed with culture medium. Finally, the labeled cells were resuspended to a concentration of 5 X 10^5^ cells/mL.

### Cytotoxicity assay

Serial dilutions of expanded Vδ2 T cells were prepared with 1 X 10^6^/mL, 5 X 10^5^/mL, 2.5 X 10^6^/mL, and 1.625 X 10^6^/mL cell concentrations. 100 μL of each dilution of PBMC enriched for Vδ2 T cells were added to 5 X 10^4^/100 μL of the Calcein AM-labeled PC3 or Huh-7 cells which resulted in effector to target cell ratios (E:T) of 20:1, 10:1, 5:1, 2.5:1, and 1.25:1; the ratio indicates numbers of enriched PBMC (Effector) and were not corrected for the percentage of Vδ2 T cells in each cell preparation. All co-cultures were performed in triplicate. The cocultures were incubated in a 96-well U-bottom plate in a 37°C cell incubator for 4 h. To account for spontaneous cell lysis, 5 X 10^4^ of the cells were cultured without Vδ2 T cells. To determine maximum cell lysis, 5 X 10^4^ cells cultured without Vδ2 T cells were completely lysed by the addition of 1% Triton-X-100 (Sigma-Aldrich) to the culture medium. After 4 h, 75 μL of supernatant from each well of the 96-well plate were transferred to a new 96-well black plate with a clear bottom and lid (VWR). Cell lysis was measured by the level of fluorescent Calcein AM detected in the supernatant using a fluorescent spectrophotometer (Biotek/Agilent, Santa Clara, CA, USA). The percentage of specific lysis was calculated with the following formula: % specific lysis = (Co-culture lysis–spontaneous lysis)÷(Maximum lysis–spontaneous lysis) X 100.

### Tumor xenograft and Vδ2 T cell treatments in mice

PC3 or Huh-7 cells were seeded in a T75 flask. When the cells were 80% confluent, they were transferred to a T175 flask. PC3 or Huh-7 cells were transduced when the cells had reached 50% confluency by replacing the medium with lentiviral vector stocks diluted in medium to result in a MOI of 5 or 10. For PC3 cells, in addition to lentivirus, polybrene was added to the cell medium at a concentration of 2 μg/mL. Three days following cell seeding, the cells were collected by trypsinization and washed twice in complete medium for inoculation into mice. For each treatment, 5-8 NSG or NRG mice were inoculated subcutaneously with 3 X 10^6^ cells/0.1 mL PC3 or Huh-7 cell plus 0.05 mL Matrigel (Corning, Corning, NY, USA) into the right flank of each mouse. Mice were weighed and tumor volumes were measured twice a week using a caliper. Tumor volume was calculated using the following equation: Tumor volume (mm^3^)=d^2^ (d=the shortest diameter) X (D/2) (D=the longest diameter). Mice were euthanized when tumor volume reached approximately 2000 mm^3^, followed by excision and weighing of the tumor. 5-8 X 10^6^ Vδ2 T cells expanded from PBMC were administered by intraperitoneal injection (IP) when the average tumor volume reached 200-300 mm^3^. Vδ2 T cells were injected by IP route every week for a total of 4 injections. ZA was administered to mice by IP injection with a dose of 100 mg/kg the day prior to each injection of Vδ2 T cells.

### Statistical analysis

The data was presented as the mean ± SEM where applicable and statistical analysis was performed with GraphPad Prism. Statistical analysis of the data from *in vivo* tumor studies was performed with the unpaired t test.

## Results

### Lentiviral vector LV-shFDPS decreases FDPS mRNA and protein expression

**Figure 1:**
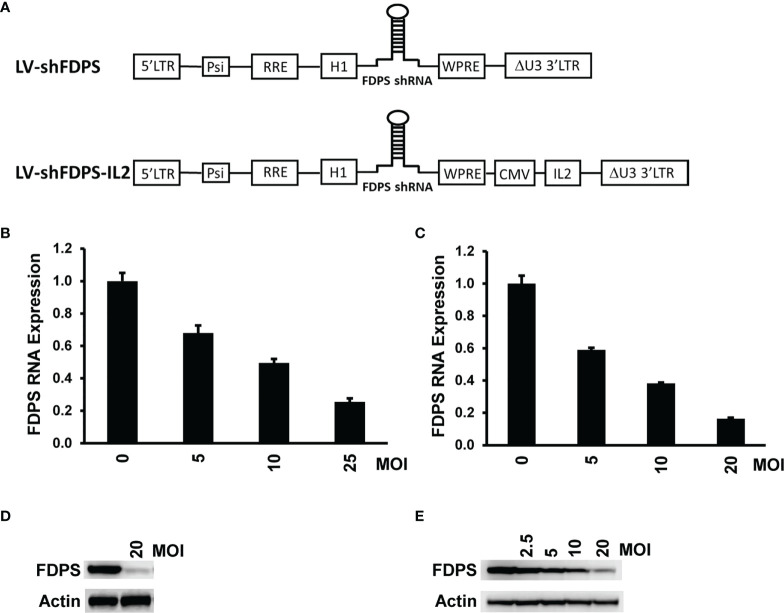
A lentivirus expressing a short-hairpin RNA (shRNA) targeting farnesyl diphosphate synthase (FDPS) reduces its expression in cancer cells. **(A)** Schematic diagram of the lentivirus vectors LV-shFDPS and LV-shFDPS-IL2. Additionally, the vectors may express the luciferase and GFP gene. **(B, C)** Real-time qPCR analysis of FDPS RNA from PC3 prostate and Huh-7 hepatocellular carcinoma cells transduced with LV-shFDPS at a MOI of 5, 10, and 20 or 25. In PC3 cells at a MOI of 25, FDPS expression was reduced by 74% and in Huh-7 cells at a MOI of 20, FDPS expression was reduced by 84%. **(D, E)** Immunoblot analysis of FDPS protein from PC3 and Huh-7 cells transduced with LV-shFDPS.

The lentiviral vector with a small hairpin (shFDPS) stem loop structure (**Figure 1A** upper panel) was used to reduce FDPS mRNA levels. The specific guide sequence used in LV-shFDPS was selected from several predicted targets after head-to-head comparisons based on mRNA levels (not shown). The ability of LV-shFDPS to reduce FDPS expression was evaluated by Real-Time qPCR or Western blot analysis in the presence of 0, 5, 10, 20 or 25 MOI of LV-shFDPS lentiviral vector in the PC3 metastatic human prostate adenocarcinoma cell line and in the Huh-7 human hepatocellular carcinoma cell line. The FDPS mRNA levels were reduced in a dose-dependent manner as compared with no LV treatment levels (100%) in PC3 cells at 5 MOI (67.9 ± 4.6%), 10 MOI (49.5 ± 2.4%), and 25 MOI (25.6 ± 2.1%) (**Figure 1B**). In Huh-7 cells, there was a decrease in FDPS mRNA at 5 MOI (59.1 ± 1.2%), 10 MOI (38.2 ± 0.6%), and 20 MOI (16.3 ± 0.7%) (**Figure 1C**). FDPS protein expression was similarly reduced in a dose-dependent manner in PC3 and Huh-7 cells, respectively (**Figures 1D, E**). These results showed that LV-shFDPS specifically targeted FDPS mRNA resulting in a reduction in both mRNA and protein levels. IL2 is an important cytokine forVδ2 T cell activation and proliferation, therefore we generated another lentiviral vector expressing both shFDPS and secretory human IL2 and showed it also reduced FDPS mRNA and protein in a dose-dependent manner in PC3 cells (**Figure 1A** lower panel; **Supplementary Figure 4**).

### Vδ2 T cell cytotoxicity against PC3 and Huh-7 cancer cell lines transduced with the lentiviral vector LV-shFDPS and treated with zoledronic acid (ZA)

**Figure 2:**
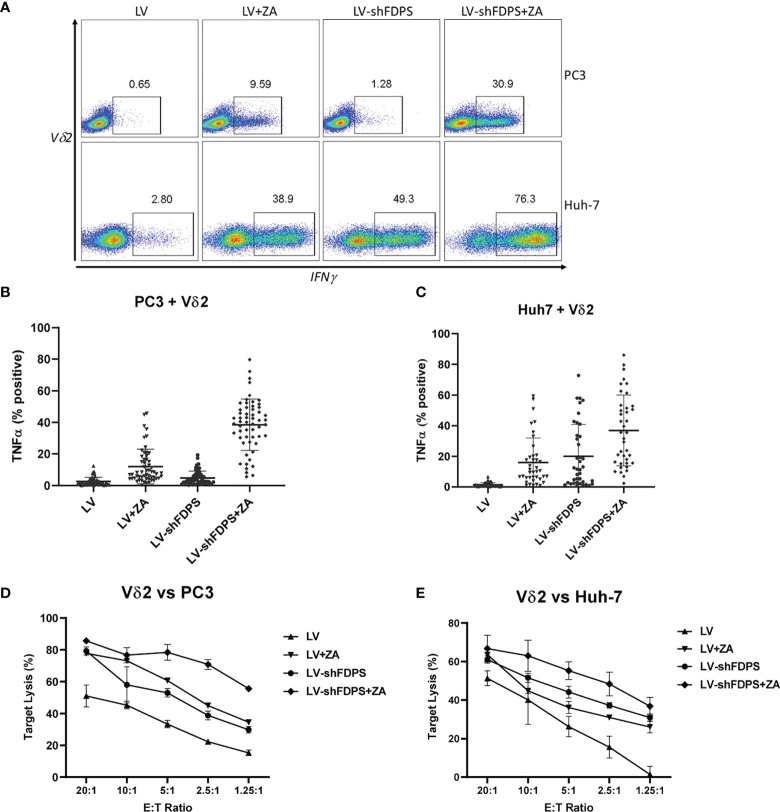
Vδ2 T cells increase cytokine production and cytotoxic activity when cultured with cancer cells treated with low-dose zoledronic acid (ZA) and transduced with shFDPS. **(A)** Representative flow cytometry dot plots from PC3 and Huh-7 cells transduced with LV-shFDPS in the presence or absence of ZA for overnight, followed by co-culture with Vδ2 T cells for 4 h. The gated region indicates the Vδ2 and IFNγ positive population. In PC3 cells, the percent positive of IFNγ producing cells was 9.59 (LV + ZA), 1.28 (LV-shFDPS), and 30.9 (LV-shFDPS + ZA). In Huh-7 cells, the percent positive of IFNγ producing cells was 38.9 (LV + ZA), 49.3 (LV-shFDPS), and 76.3 (LV-shFDPS + ZA). **(B, C)** LV-shFDPS transduced PC3 and Huh-7 cells were treated either with or without ZA for overnight, followed by co-culture with Vδ2 T cells for 4 h. The cells were analyzed by flow cytometry for Vδ2 and TNFα expression. The data represents a summary of assays using Vδ2 T cells from multiple donors (PC3, N=56; Huh-7, N=39). Each dots represents the percentage of Vδ2 and TNFα positive cells from an individual donor. In PC3 cells, the percent positive of TNFα producing cells was 12 ± 10.9% (LV + ZA), 4.8 ± 4.3% (LV-shFDPS), and 38.6 ± 16.3% (LV-shFDPS + ZA). In Huh-7 cells, the percent positive of TNFα producing cells was 16.1 ± 15.9% (LV + ZA), 20 ± 10.8% (LV-shFDPS), and 36.9 ± 23.1% (LV-shFDPS + ZA). **(D, E)** LV or LV-shFDPS transduced PC3 and Huh-7 were treated either with or without ZA for overnight as the target cells and labeled with calcein AM. This was followed by culturing the target cells with a serial dilution of Vδ2 effector cells in target ratios (E/T) for 4 h. All co-cultures were performed in triplicate. Cell lysis was measured by the level of fluorescent calcein AM detected in the supernatant.

To determine whether transduction of cancer cells with the lentiviral vector LV-shFDPS can activate Vδ2 T cells, we measured cytokine production and cell lysis by Vδ2 T cells co-cultured with LV-shFDPS transduced PC3 and Huh-7 cells. Activation of Vδ2 T cells was indicated by increased production of the proinflammatory cytokines IFNγ or TNFα. PC3 or Huh-7 cells were transduced with either LV-shFDPS or LV-Control (LV) for 48 h followed by ZA (1 μM) treatment for 24 h. The ZA dose of 1 μM was used because this is in the range of vivo bioavailability (50). The cell lines were co-cultured with Vδ2 T cells for 4 h and analyzed subsequently for Vδ2 and TNFα expression. By gating IFNγ positive cells among the Vδ2 population, activation of Vδ2 T cells was indicated by IFNγ expression. In PC3 cells, the percentage of IFNγ producing cells was 9.59 (LV (control vector) + ZA), 1.28 (LV-shFDPS) and 30.9 (LV-shFDPS + ZA) (**Figure 2A** upper panel).

In Huh-7 cells, the percent positive of IFNγ producing cells was 38.9 (LV + ZA), 49.3 (LV-shFDPS), and 76.3 (LV-shFDPS + ZA) (**Figure 2A** lower panel). Donor-specific variation was controlled by testing expanded Vδ2 T cells (60-80%) from multiple, unrelated donors. The activity of LV-shFDPS and ZA treatment was evaluated by co-culture of Vδ2 T cells with PC3 and Huh-7 cells for 4 h, followed by flow cytometry analysis for Vδ2 and TNFα expression. The data represents a summary of assays using Vδ2 T cells from multiple donors (PC3, N=56; Huh-7, N=39). Each dot represents the percentage of Vδ2 and TNFα positive cells from an individual donor. In PC3 cells, the average percent positive of TNFα producing cells was 12 ± 10.9% (LV + ZA), 4.8 ± 4.3% (LV-shFDPS), and 38.6 ± 16.3% (LV-shFDPS + ZA) (**Figure 2B** **)**. In Huh-7 cells, the average percentage of cells positive for TNFα expression was 16.1± 15.9% (LV + ZA), 20 ± 10.8% (LV-shFDPS), and 36.9± 23.1% (LV-shFDPS + ZA) (**Figure 2C**). The results show that treatment of PC3 and Huh-7 cells with either LV-shFDPS or ZA alone was insufficient to fully activate Vδ2 T cells and the highest levels of cell activation occurred with the combined treatments. Similar results were seen with HepG2, MDA-MB-231, MiaPaCa, A549, and FaDu cells (**Supplementary Figure 2** **).**

The cytolytic activity of Vδ2 T cells by LV-shFDPS and ZA treatment of PC3 and Huh-7 cells was tested using a Calcein AM assay. PC3 or Huh-7 cells were treated with LV-shFDPS or ZA alone or in combination and then loaded with Calcein AM before incubating with Vδ2 T cells in various effector to target ratios (E: T) for 4 h. The supernatant from these co-cultures was evaluated for the presence of Calcein AM, which is indicative of tumor cell lysis. The results were analyzed, and the percent specific lysis values were calculated. At an E:T ratio of 2.5:1 for Vδ2 T cells to PC3 cells, the percent lysis was 22.2 ± 1.1% (LV), 45.1 ± 1.4% (LV + ZA), 38.8 ± 2.8% (LV-shFDPS), and 70.9 ± 3.1% (LV-shFDPS + ZA) (**Figure 2D**). At an E:T ratio of 2.5:1 for Vδ2 T cells to Huh-7 cells, % lysis was 15.6 ± 5.6% (LV), 31.0 ± 1.0% (LV + ZA), 37.3 ± 1.4% (LV-shFDPS), and 48.4 ± 6.1% (LV-shFDPS + ZA) (**Figure 2E**). Vδ2 T cells showed the highest cytokine production and cytotoxicity against PC3 and Huh-7 cells treated with both LV-shFDPS and ZA as compared with each alone.

### The effect of Vδ2 T cells on PC3 tumors modified by the lentiviral vector LV-shFDPS in immunodeficient mice

**Figure 3:**
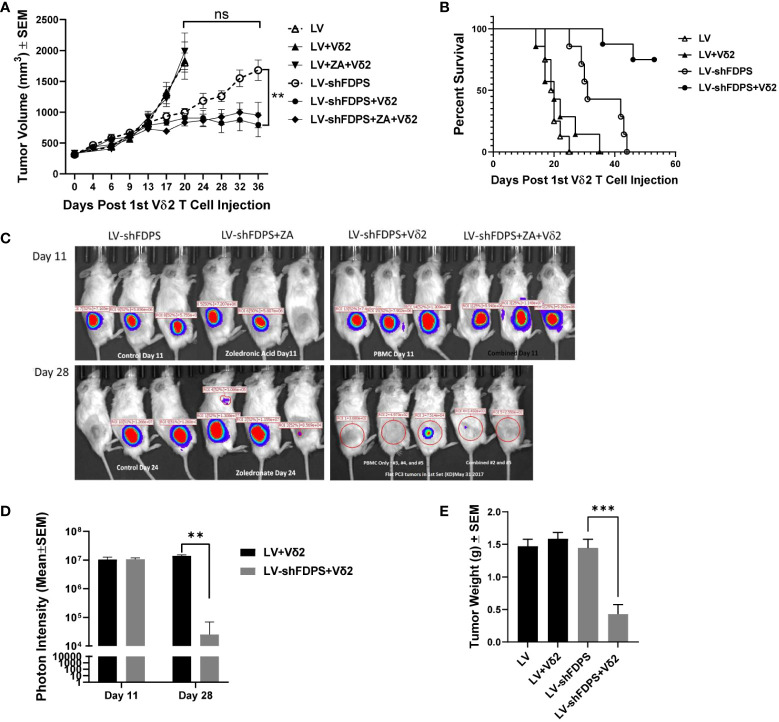
Vδ2 T cells suppress the growth of PC3 prostate carcinoma tumors transduced with a lentivirus expressing a shRNA targeting FDPS. **(A)** Vδ2 T cells significantly suppressed the growth of PC3 tumors transduced with LV-shFDPS as compared with LV (**p<0.0037, N=8). At the end of the study, 36 days post Vδ2 injection, the average tumor volume for LV-shFDPS was 1680 ± 166 mm^3^ which was reduced to 795 ± 193 mm^3^ when combined with Vδ2 treatment. There was minimal effect on tumor volume with ZA treatment with or without LV-shFDPS. **(B)** An analysis using a Kaplan-Meier survival curve was based on the end event being when the tumor size reached 2000 mm^3^. There was a significant survival advantage in mice with PC3 tumors transduced with LV-shFDPS and treated with Vδ2 T cells as compared with no Vδ2 T cells (****p < 0.0001, N=8). All mice with LV transduced PC3 tumors with or without treatment of Vδ2 T cells were sacrificed up to day 22. In mice with LV-shFDPS transduced PC3 tumors, mice survived up to day 44, and with Vδ2 T cell treatment all mice survived up to day 60. **(C, D)** Mice were imaged for luciferase expression with a Xenogen IVIS200 bioluminescent imager. All tumor groups showed a similar photon intensity 11 days after the initial injection of Vδ2 T cells. On day 28, in the mice injected four times with Vδ2 T cells, the photon intensity of LV-shFDPS transduced PC3 tumors as compared with LV was significantly decreased from 1.4 X 10^7^ to 2.5 X 10^4^ photon units (** *p*=0.002, N=6). **(E)** Comparison of LV and LV-shFDPS transduced PC3 tumors in combination with Vδ2 T cell treatment. At the end of the study, each group of mice (N=7 or 8) were euthanized, tumors were extracted and weighed. In mice injected four times with Vδ2 T cells, the tumor weight of LV-shFDPS transduced PC3 tumors was significantly decreased as compared with LV from 1.5 to 0.5 g (*** *p*=0.0002, N=8). ns, not significant.

A xenotransplant mouse tumor model using NSG immunodeficient mice was used to evaluate the anti-tumor activity of Vδ2 T cells against PC3 tumors transduced with LV-shFDPS at an MOI of 5. PC3 cells were injected into the flanks of mice and grown to a size of 200-300 mm^3^. Expanded Vδ2 T cells (6-10 X 10^6)^ were injected weekly by intraperitoneal (IP) delivery. One group of mice additionally received ZA injections also given by IP. Mice were monitored for health and tumor volume was measured twice weekly until conclusion of the study. LV-shFDPS transduced tumors grew slower than the LV-Control (LV) tumors, but at the end of the study reached a similar average tumor volume which was not significantly different (ns *p*=0.5967, N=8) (**Figure 3A**). PC3 cells transduced with LV-shFDPS significantly suppressed tumor growth with or without ZA treatment after Vδ2 T cell injections (* *p*=0.0155, ** *p*=0.0037, N=8). At the end of the study, 36 days post 1^st^ Vδ2 injection, the average tumor volume for LV-shFDPS was 1680 ± 166 mm^3^ which was reduced to 795 ± 193 mm^3^ when combined with Vδ2 treatment (**Figure 3A**). Therefore, LV-shFDPS alone decreased the growth of PC3 tumors, but this was further increased when combined with Vδ2 T cells.

A Kaplan Meier survival analysis was done with the end point being the number of days after the initial injection of Vδ2 T cells before the tumor reached 2000 mm^3^. There was a significant survival advantage in mice with PC3 tumors transduced with LV-shFDPS and treated with Vδ2 T cells as compared with no Vδ2 T cells (**** *p*<0.0001, N=8) (**Figure 3B**). All mice with LV (control) transduced PC3 tumors with or without treatment of Vδ2 T cells had been euthanized by day 22. In mice implanted with LV-shFDPS transduced PC3 tumors, mice survived to day 44, and with Vδ2 T cell treatment all mice were still alive on day 60 (**Figure 3B**).

Since the lentiviral vectors used in this mouse study also carried the firefly luciferase gene, bioluminescence imaging could be used to monitor tumor growth. Bioluminescence images of LV-shFDPS transduced PC3 tumors were captured on days 11 and 28 after the initial injection of Vδ2 T cells (**Figure 3C**). By day 11, mice had been injected twice with Vδ2 T cells but there were no differences in photon levels between the LV and LV-shFDPS tumor groups. On day 28, the mice had been injected 4 times with Vδ2 T cells and there was a significant reduction in photon levels from 1.4 X 10^7^ to 2.5 X 10^4^ in the LV tumors compared with tumors bearing the LV-shFDPS (** *p*=0.002, N=6) (**Figure 3D** **)**.

At the end of the study, all mice were sacrificed when PC3 tumors reached 2000 mm^3^; tumors were collected and weighed. There were significantly lower weights for the LV-shFDPS tumors; from 1.5 to 0.5 g (3-fold decrease) in mice treated with Vδ2 T cells (*** *p*=0.0002, N=8) (**Figure 3E**). The results demonstrated that treatment of PC3 tumors with LV-shFDPS and Vδ2 T cells lead to PC3 tumor growth suppression. Tumors were also analyzed by flow cytometry for the presence of Vδ2 T cells. Vδ2 T cells were detected in both LV-shFDPS transduced tumors after an injection of 8 million Vδ2 T cells (**Supplementary Figure 3** **).**

### Effects of Interleukin-2 (IL2) on the anti-tumor activity of Vδ2 T cells against PC3 tumors modified by the lentiviral vector LV-shFDPS in immunodeficient mice

**Figure 4:**
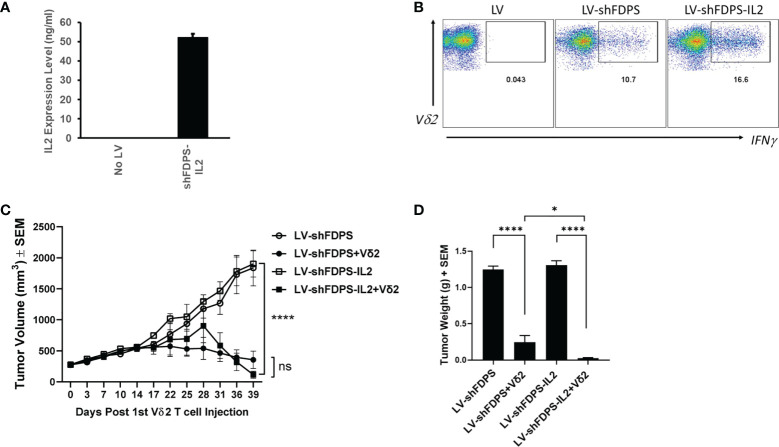
Vδ2 T cells suppress the growth of PC3 prostate carcinoma tumors transduced with a lentivirus expressing IL-2 and a shRNA targeting FDPS. **(A)** ELISA analysis of secreted IL2 expression by PC3 cells transduced with LV-shFDPS-IL2. **(B)** Representative flow cytometry dot plots from PC3 cells transduced with LV-shFDPS or LV-shFDPS-IL2, followed by co-culture with Vδ2 T cells for 4 h. The gated region indicates the Vδ2 and IFNγ positive population. The percent positive of IFNγ producing cells was 10.7 (LV-shFDPS) and 16.6 (LV-shFDPS-IL2). **(C)** Tumor volume of PC3 cells transduced with LV-shFDPS or shFDPS-IL2. In mice treated with Vδ2 T cells, the tumor volume of PC3 cells transduced with LV-shFDPS was significantly decreased at the end of the study from 1900 mm^3^ to 356 mm^3^ and with LV-shFDPS-IL2 from 1835 mm^3^ to 116 mm^3^ (****p=0.0001, ***p=0.001, N=8). There was no significant difference when comparing LV-shFDPS and LV-shFDPS-IL2 (ns *p*=0.743, N=8). **(D)** Comparison of LV, LV-shFDPS, and LV-shFDPS-IL2 transduced PC3 tumors in combination with Vδ2 T cell treatment. At the end of the study, each group of mice (N=8) were euthanized, tumors were extracted and weighed. There was a significant decrease in tumor weight of PC3 tumors transduced with LV-shFDPS when treated with Vδ2 T cells from 1.25 g to 0.23 g (5.5-fold decrease, ****p<0.0001, N=8). The addition of IL2 in the LV-shFDPS vector also significantly decreased tumor weight in mice treated with Vδ2 T cells as compared with LV-shFDPS-IL2 alone from 1.31 g to 0.04 g. There was also a significant 6-fold decrease in the tumor weight of LV-shFDPS-IL2 vs LV-shFDPS groups with Vδ2 T cells (* *p*=0.012, N=8). ns= not significant.

IL2 is used frequently to increase the activation and proliferation of Vδ2 T cells. We tested if adding an IL2 gene to LV-shFDPS and expressing both in a tumor cell line, would increase Vδ2 T cell activity in a coculture assay. PC3 cells were transduced with 5 MOI of LV-shFDPS-IL2 lentiviral vector and secreted IL2 was detected in cell culture medium by ELISA. The mean concentration of IL2 in culture medium was 52 ng/mL from 4 replicates as determined with a standard curve (**Figure 4A**). Next, we determined if LV-shFDPS-IL2 produced active IL2. We compared LV-shFDPS and LV-shFDPS-IL2 transduced PC3 cells for the ability to stimulate IFNγ expression in co-cultured Vδ2 T cells. The frequency of Vδ2 T cells expressing IFNγ was higher when tumor cells were cultured with LV-shFDPS-IL2 (16.6%) compared to tumor cells transduced with LV-shFDPS (10.7%) (**Figure 4B**).

Expressing IL2 from LV-shFDPS transduced PC3 tumors impacted the growth of tumors in NSG mice treated with Vδ2 T cells. Mice were inoculated with 3 X 10^6^ LV-shFDPS or LV-shFDPS-IL2 transduced PC3 cells. Once tumor volumes reached 200-300 mm^3^, each group of mice were distributed into two subgroups. One subgroup (N=8) was injected intraperitoneally with 6-10 X 10^6^ Vδ2 T cells and the other group was injected with vehicle (N=8). One group of mice was injected with Vδ2 T cells once a week for a total of four weeks. Mouse body weights and tumor volumes were measured twice a week and the animals were euthanized when tumors reached 2000 mm^3^ or at the end of the study. At the end of the study, 39 days after the 1^st^ Vδ2 T cell injection, the average tumor volume for LV-shFDPS and LV-shFDPS-IL2 was 1835 ± 289 and 1900 ± 210 mm^3^ which was reduced to 356 ± 140 mm^3^ and 116 ± 55 mm^3^ when combined with Vδ2 T cell treatment, respectively (**Figure 4C**). In mice treated with Vδ2 T cells, the growth of PC3 tumors transduced with LV-shFDPS or LV-shFDPS-IL2 were significantly suppressed compared with mice that did not receive Vδ2 T cells (**** *p*=0.0001, *** *p*=0.001, N=8) (**Figure 4C**). However, there was no significant difference in tumor volume when comparing LV-shFDPS and LV-FDPS-IL2 (ns *p*=0.743, N=8) (**Figure 4C**). Tumor weights were determined for excised tumors collected during necropsy. There was a significant decrease in tumor weight for the LV-shFDPS vs LV-shFDPS + Vδ2 groups from 1.25 to 0.23 g (5.5-fold decrease) and 1.31 to 0.04 g (32-fold decrease) for the LV-shFDPS-IL2 vs LV-shFDPS-IL2 + Vδ2 groups (**** *p*<0.0001, **** *p*<0.0001, N=8) (**Figure 4D**). There was also a significant 6-fold decrease in the tumor weight of LV-shFDPS-IL2 + Vδ2 vs LV-shFDPS + Vδ2 groups (* *p*=0.012, N=8) (**Figure 4D**). Therefore, IL2 enhanced Vδ2 T cell activity and suppressed the growth of PC3 tumors when FDPS expression was reduced by shRNA. Notably, 4 out of 8 mice bearing LV-shFDPS-IL2 modified tumors lost weight and died 3 weeks after the initial injection of Vδ2 T cells. The cause of death was suspected to be a form of tumor lysis syndrome, but the pathology was not definitive. Tumor volume measurements for the mice that died were included up to the point of death and only 4 surviving mice to the end of the study were included in the tumor weight measurements.

### Effects of Vδ2 T cells on LV-shFDPS transduced Huh-7 tumors in immunodeficient mice

**Figure 5:**
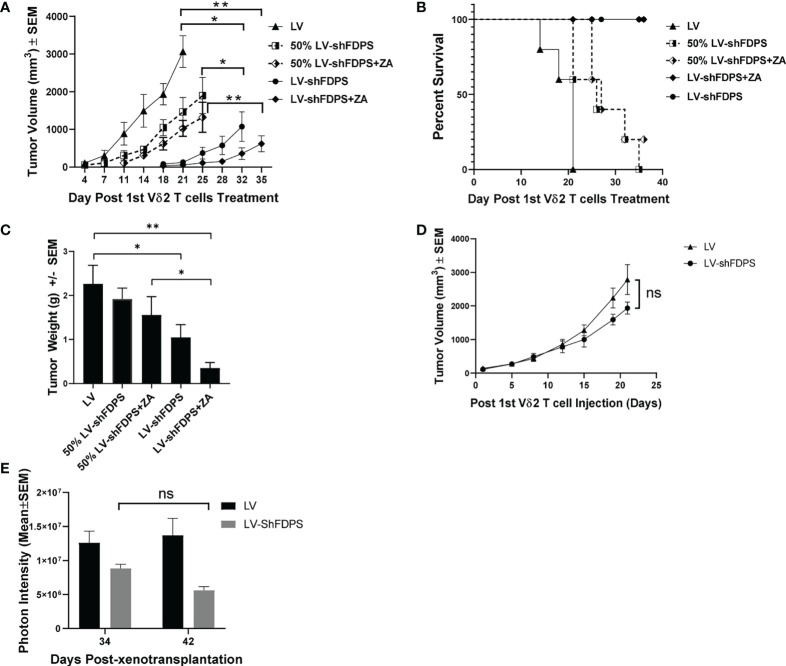
Vδ2 T cells suppress the growth of Huh-7 hepatocellular tumors transduced with a lentivirus expressing a shRNA targeting FDPS. **(A)** Tumor volume in mice with LV, 100% LV-shFDPS or 50% LV-shFDPS transduced Huh-7 cells in the presence or absence of ZA. The tumor volume of 100% LV-shFDPS and 50% LV-shFDPS tumors was decreased as compared with LV tumors in the presence or absence of ZA. There was a significant decrease in tumor volume in mice with 100% LV-shFDPS and treated with ZA or without ZA as compared with LV tumors (** *p*=0.001, *p=0.014, N=5). The tumor volumes in mice implanted with 100% LV-shFDPS transduced cells were significantly lower than in mice who received 50% transduced cells (*p=0.024, N=5). **(B)** A Kaplan-Meier survival curve of mice which were transplanted with LV, LV-shFDPS, or 50% LV-shFDPS transduced Huh-7 cells in the presence or absence of ZA. Each group of mice (N=5) was euthanized when tumors reached 2000 mm^3^. The survival of mice with LV transduced tumors was reduced versus mice with LV-shFDPS and 50% LV-shFDPS transduced tumors in the presence or absence of ZA. There was a significant survival advantage for mice with Huh-7 tumors transduced with LV-shFDPS as compared with LV (****p<0.0001, N=5). Additionally, there was a significant survival advantage for mice with 100% versus 50% LV-shFDPS transduced tumors in the presence or absence of ZA. (**p=0.0089, **p=0.0048, N=5). **(C)** Tumor weight of mice with LV, 100% LV-shFDPS or 50% LV-shFDPS transduced Huh-7 cells in the presence or absence of ZA. Each group of mice (N=5) were euthanized and tumors were extracted and weighed. The tumor weight of 100% LV-shFDPS and 50% LV-shFDPS tumors was decreased versus LV tumors in the presence and absence of ZA. The tumor weights of the 100% LV-shFDPS and 100% LV-shFDPS + ZA group were significantly decreased by 2.3 and 5.75-fold, respectively, compared with LV (* *p*=0.045, ** *p*=0.003, N=5). The tumor weights of the 100% LV-shFDPS + ZA group also showed a significant decrease compared with 50% LV-shFDPS + ZA (* *p*=0.0237, N=5). **(D)** Volume of Huh-7 tumors in mice injected intratumorally with LV and LV-shFDPS. At the end of the study, 22 days post Vδ2 injections, the tumor volume decreased from 2785 ± 181 mm^3^ to 1934 ± 447 mm^3^ in LV versus LV-shFDPS injected tumors (ns *p*=0.108, N=8). **(E)** Mice were imaged for luciferase expression with a Xenogen IVIS200 bioluminescent imager. The photon intensity of tumors injected with LV was not significantly different from day 34 to 42 after treatment with Vδ2 T cells, whereas there was a decrease in the photon intensity of tumors injected with LV-shFDPS from 8.8 X 10^6^ to 5.6 X 10^6^ from day 34 to 42 (ns *p*=0.107, N=8). ns, not significant.

Next, we examined the effect Vδ2 T cells have on another xenotransplant model, this time using Huh-7 hepatocellular carcinoma tumor cells transduced with LV-shFDPS and implanted in NRG mice. The Huh-7 cells were transduced with either LV or LV-shFDPS and cells were injected into the flanks of NRG mice in the following numbers: 2 X 10^6^ LV (N=10), 2 X 10^6^ LV-shFDPS (N=10), or a 1:1 mixture of 1 X 10^6^ LV + 1 X 10^6^ LV-shFDPS (N=10; designated 50% transduced mice). A week after injection, all mice were injected once a week with 7 X 10^6^ Vδ2 T cells by IP delivery and half of the mice received ZA treatment for 5 weeks. There was a significant decrease in tumor volume in mice with 100% LV-shFDPS transduced cells and treated with ZA as compared with LV transduced cells (** *p*=0.001, N=5) (**Figure 5A**). Additionally, there was a significant decrease in the volume of tumors containing 100% LV-shFDPS transduced cells without ZA as compared with LV transduced cells (* *p*=0.014, N=5) (**Figure 5A**). The tumor volumes in mice implanted with 100% LV-shFDPS transduced cells were significantly lower than in mice who received 50% transduced cells (* *p*=0.024, N=5) (**Figure 5A**). The Kaplan Meier survival curve was based upon the event when tumors reached 2000 mm^3^. There was a significant survival advantage for mice with Huh-7 tumors transduced with LV-shFDPS as compared with LV in the presence of Vδ2 T cells (**** *p*<0.0001, N=5). Additionally, there was a significant survival advantage for mice with 100% versus 50% LV-shFDPS transduced tumors in the presence or absence of ZA. (** *p*=0.0089, ** *p*=0.0048, N=5). The survival curve showed that mice with LV transduced tumors were sacrificed 21 days after the initial injection of Vδ2 T cells and mice with 100% and 50% LV-shFDPS transduced tumors remained alive at the end of the study on day 37 (**Figure 5B**). At the end of the study, 23 days following the initial Vδ2 T cell injections, mice were euthanized, and tumors were removed and weighed. The average tumor weights were 2.3 ± 0.4 g (LV), 1.9 ± 0.2 g (50% LV-shFDPS) 1.6 ± 0.4 g (50% LV-FDPS + ZA), 1.0 ± 0.3 g (100% LV-shFDPS), and 0.4 ± 0.1 g (100% LV-FDPS + ZA) (**Figure 5C**). The tumor weights of the 100% LV-shFDPS and 100% LV-shFDPS + ZA group were significantly decreased by 2.3 and 5.75-fold, respectively, compared with LV (* *p*=0.045, ** *p*=0.003, N=5) (**Figure 5C**). The tumor weights of the 100% LV-shFDPS + ZA group also showed a significant decrease compared with 50% LV-shFDPS + ZA (* *p*=0.0237, N=5) (**Figure 5C**). In rough terms, the therapeutic effects among animals with 50% transduced tumors were half the levels observed for animals with 100% uniformly transduced tumors.

### Effects of Vδ2 T cells on Huh-7 tumors by intra-tumoral (IT) injection of LV-shFDPS

The possibility of using a lentiviral vector for *in vivo* treatment of human cancer will depend on the efficiency of IT injection. To mimic the clinical procedure, we performed a pilot experiment using intratumoral (IT) injection of LV-shFDPS in Huh-7 tumors in combination with Vδ2 T cells. NSG mouse were injected subcutaneously with 2 X 10^6^ Huh-7 cells in the right flank. Three weeks after injection, the average tumor sizes ranged from 100-900 mm^3^; mice were placed into two groups with roughly equal distribution of starting tumor sizes. One group of tumors (N=8) were injected daily with 60 μL of LV and the other group (N=8) with 60 μL of LV-shFDPS for three consecutive days. Five days after IT injection of LV or LV-shFDPS, mice were injected with 7 X 10^6^ Vδ2 T cells twice a week for a total of 4 weeks. Although the LV-shFDPS tumors (1934 ± 181 mm^3^) grew slower than LV tumors (2785 ± 447. mm^3^), the overall tumor volume showed no significant differences between LV and LV-shFDPS at the end of the study (ns *p*=0.108, N=8) (**Figure 5D**). It may be due to an insufficient amount of LV-shFDPS injected into the tumors that the effect of treatment on tumor growth with Vδ2 T cells was limited. Since LV and LV-shFDPS contain the luciferase gene, the injected lentiviral vector could be detected by bioluminescence imaging. Bioluminescent imaging demonstrated that tumors injected with LV-shFDPS showed an average decrease in photon intensity from 8.8 X 10^6^ to 5.6 X 10^6^ from day 34 to day 42 but this was not significant (ns *p*=0.107, N=8) (**Figure 5E**).

## Discussion

A distinguishing feature of human Vδ2 T cells is the capacity for T cell receptor-dependent sensing of metabolic changes in malignant tissues, specifically the upregulation of mevalonate pathway flux signaled by increased levels of IPP. Several groups have appreciated the potential for exploiting the unique specificity of Vδ2 T cells for cancer therapy. Here, we explored the potential for enhanced recognition and destruction of tumors following direct genetic manipulation of tumor cells to increase IPP levels. We demonstrated significantly enhanced potency of Vδ2 T cells from healthy donors for suppressing growth of genetically modified as compared to unmodified tumor cells *in vivo*. We further demonstrated the dose dependence of Vδ2 T cell tumor suppression, by varying the proportion of tumor cells expressing shFDPS needed to increase IPP levels. The inefficient suppression of tumor growth after intratumoral injection of the lentivirus vector may be a further demonstration of dose dependency, although the pilot studies presented here are not conclusive. Lastly, we confirmed that dual targeting of FDPS activity, by reducing mRNA and protein levels plus adding the competitive enzyme inhibitor zoledronic acid, increased the potency for Vδ2 T cells suppression of tumor cell growth. These findings provide insight into the possibility of combining genetic and small molecule therapies with, *ex vivo* expanded Vδ2 T cells, in a cancer therapy regimen.

There are several limitations to these studies. While group sizes were generally sufficient at 8-10 mice per group, we did not explore a broad range of mixed tumors (cells with or without shFDPS expression) and definitive conclusions about the relationship between vector dose and tumor suppression are not warranted at this time. Experiments with lentivirus vectors expressing both shFDPS and IL2 were limited in numbers and the precise cause of animal deaths was uncertain, although we note that the vector construct was not toxic as animal deaths occurred only after infusing Vδ2 T cells. In this context we did not test mixtures of individual vectors (LV-shFDPS + LV-shFDPS-IL2 in varying ratios) to see whether a lower proportion of IL2 producing tumor cells would support Vδ2 T cells effects without the associated toxicity. Finally, the intratumoral injection pilot studies are likely to require multiple repetitions and perhaps alternate delivery sites or methods.

Our key findings are that genetic manipulation of tumor cells increases the potential for Vδ2 T cell activation and tumor suppression *in vivo*. Tumor suppression occurred in the absence of IL-2 treatment, although co-expressing IL-2 with shFDPS on the same vector appears to have increased potency. Dual targeting of FDPS activity, using LV-shFDPS plus ZA increased tumor suppression but the added benefit of adding ZA was modest compared to what was observed for dual targeting *in vitro*.

Several published studies explored the effects of N-bisphosphonate (NBP) treatment in combination with Vδ2 T cells in mouse models. For example, Kabelitz et al. tested whether NBP alendronate plus IL2 suppressed the growth of the melanoma cell line MeWo in NSG mice treated with Vδ2 T cells (46). Santolaria et al. tested the NBP pamidronate plus Vδ2 T cells on the prostate adenocarcinoma cell line PC3 in NSG mice (51). These studies demonstrated decreased tumor growth when Vδ2 T cells were administered to NBP treated mice and the effect was greater with repeated treatment of both NBP and Vδ2 T cells. The therapeutic effect of Vδ2 T cells on LV-shFDPS modified PC3 or Huh-7 tumors was at least as potent as treating mice with Vδ2 T cells and alendronate plus IL-2 (46) or pamidronate (51). In most cases, mouse survival was better and tumor weights were reduced more in mice with LV-shFDPS modified tumors treated with Vδ2 T cells; with or without the NBP zoledronic acid had little impact on tumor growth and mouse survival.

The cytokine IL2 stimulates expansion and Vδ2 T cell cytotoxic activity. We observed that PC3 cells transduced with LV-shFDPS-IL2 as compared with LV-shFDPS stimulated more IFNγ production. Additionally the growth of PC3 tumors with LV-shFDPS-IL2 was decreased as compared with LV-FDPS in combination with Vδ2 T cells. However, we observed weight loss in some mice with LV-FDPS-IL2 transduced tumors, which may be caused by a supraphysiological level of circulating IL2 in the mice. In mice injected with a mixture of cells, 50% LV-FDPS-IL2 + 50% LV, there was no weight loss or deaths observed (data not shown). This suggests that the optimum level of IL2 can enhance the activity of Vδ2 T cell activity without causing toxicity. In the current vector, IL2 expression is being controlled by the strong CMV promoter, but weaker promoters or promoter regulatory units could be engineered into the vector for future studies.

Several groups have conducted exploratory clinical trials to evaluate the potential for activating Vδ2 T cells for cancer therapy. Zoledronic acid plus IL-2 therapy was tested in male patients with hormone-refractory prostate cancer (23). A similar approach was used to treat patients with advanced renal cell carcinoma (52) along with many other studies summarized in recent reviews. Recent reports evaluated Vδ2 T cell therapies providing positive outcomes in late-stage lung or liver cancer (53), treatment refractory lung cancer (54), cholangiocarcinoma (55), and gastric carcinoma (56). A meta-analysis of 13 clinical trials involving Vδ2 T cell therapy, either adoptive transfer of ex vivo expanded Vδ2 T cells or *in vivo* activation of these cells using PAg or bisphosphonate compounds, showed a significant event rate for Vδ2 T cell therapies and no significant association with severe adverse events (57). These studies affirm an overall view that Vδ2 T cell therapies involving ex vivo or *in vivo* cell activation and expansion, can provide positive therapeutic benefit but overall have modest potency.

Our approach was to improve upon these preclinical results by genetically targeting FDPS to further increase IPP levels in tumor cells and promote their subsequent destruction by Vδ2 T cells (41). Thus, we are exploring the utility of a new tool for manipulating the Vδ2 T cell capacity for tumor suppression and believe it can be combined with PAg or bisphosphonate stimulation, IL-2 treatment and ex vivo expanded autologous or allogeneic effector cells in a continuing effort to develop potent treatment strategies.

Several major obstacles must be overcome to realize the potential for gamma delta T cell-based cancer therapy. Use of bisphosphonates drugs for *in vivo* stimulation of Vδ2 T cells is inefficient, possibly due to the unusual pharmacodynamics of these drugs which precipitate in bone (58, 59) and cytokine supplementation, using IL-2 or others, has inherent toxicity. Activating Vδ2 T cells by targeting butyrophilin complexes on tumor cell membranes is an area of intense interest (60, 61) and has potential for combining with a genetic approach to achieve higher potency for tumor cell killing. The value for genetic modification of tumor cells was demonstrated in our studies but we recognize the potential for alternative approaches based on the same underlying concept of decreasing FDPS enzyme activity in tumor cells. Alternate approaches might involve the use of tumor-specific oncoviruses expressing the shRNA specific for FDPS, and direct delivery of shRNA to tumor cells is another possibility for increasing treatment potency. By modifying tumor cells through multiple, concurrent methods including genetic modification, drug treatment and agonist antibodies or antibody-like molecules, we hope to increase potency and fully operationalize the power of Vδ2 T cells and their capacity for broad recognition of multiple tumor types.

## Data availability statement

The raw data supporting the conclusions of this article will be made available by the authors, without undue reservation.

## Ethics statement

The animal study was reviewed and approved by University of Maryland School of Medicine-Institutional Review Board.

## Author contributions

M-LL is the first author of the manuscript, designed, performed, and analyzed the *in vivo* studies and cellular assays, and wrote the initial version of the paper; TL is a shared first author of the manuscript, supervised the project, contributed to molecular and cellular assays, data analysis, and revision and preparation of the manuscript; HL contributed to study design and cellular assays; LX contributed to molecular assays; CDP supervised the project and contributed to revising the main text of the manuscript; TL is the corresponding author of the manuscript. All authors contributed to the article and approved the submitted version.

## Conflict of interest

The authors M-LL, TL, and LX are employed by American Gene Technologies and HL and CDP are former employees of American Gene Technologies and currently employed by Viriom, Inc.

## Publisher’s note

All claims expressed in this article are solely those of the authors and do not necessarily represent those of their affiliated organizations, or those of the publisher, the editors and the reviewers. Any product that may be evaluated in this article, or claim that may be made by its manufacturer, is not guaranteed or endorsed by the publisher.
