# Supplementary material for: Reducing farnesyl diphosphate synthase levels activates Vγ9Vδ2 T cells and improves tumor suppression in murine xenograft cancer models
Source: Front Immunol. 2022 Oct 5;13:1012051. doi: 10.3389/fimmu.2022.1012051 (PMC9581136; doi:10.3389/fimmu.2022.1012051)
Supplement: Supplementary file 1 [file DataSheet_1.docx]

**
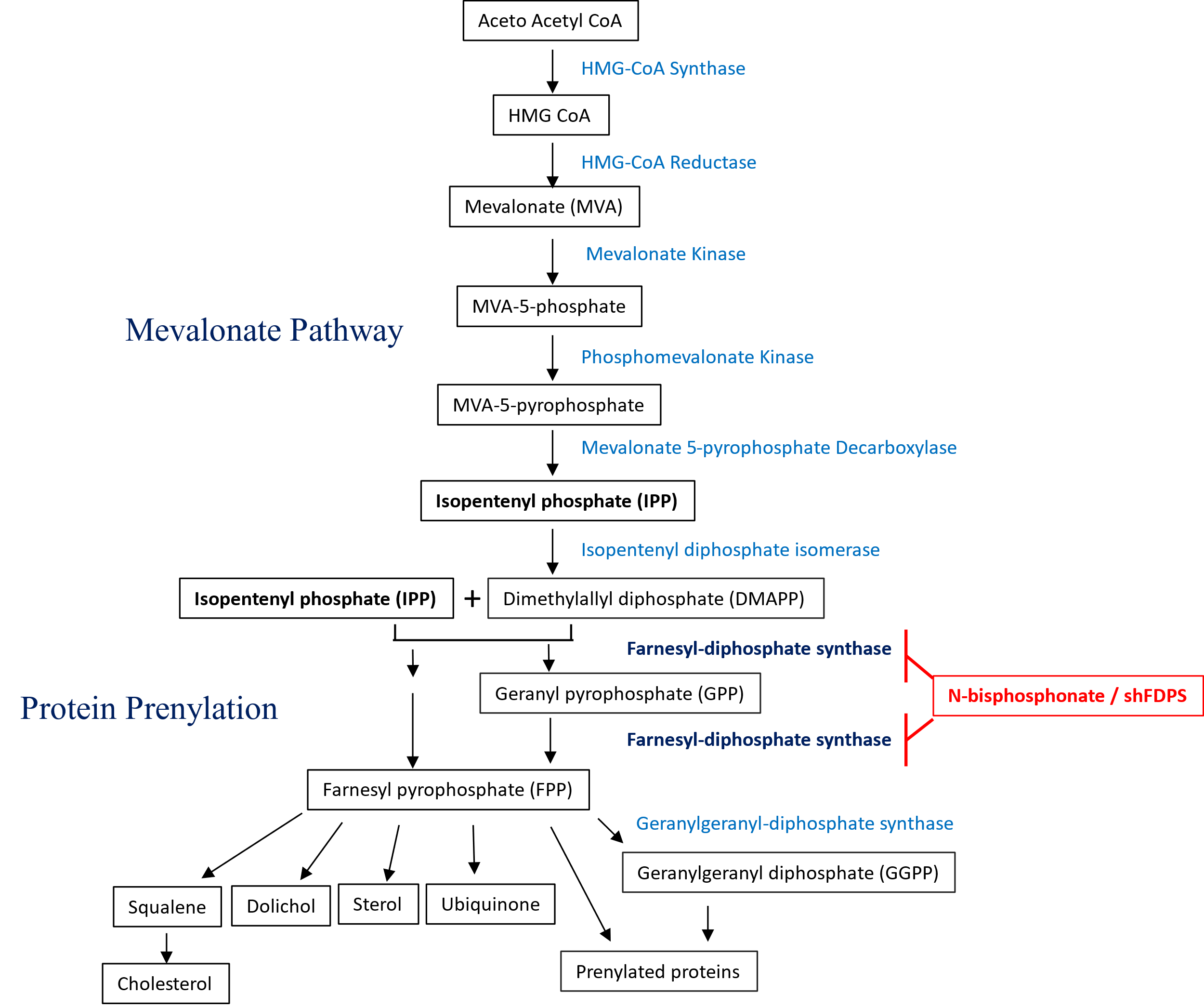
**

**Supplementary Figure 1**. Diagram of the mevalonate pathway showing inhibition of FDPS.

**
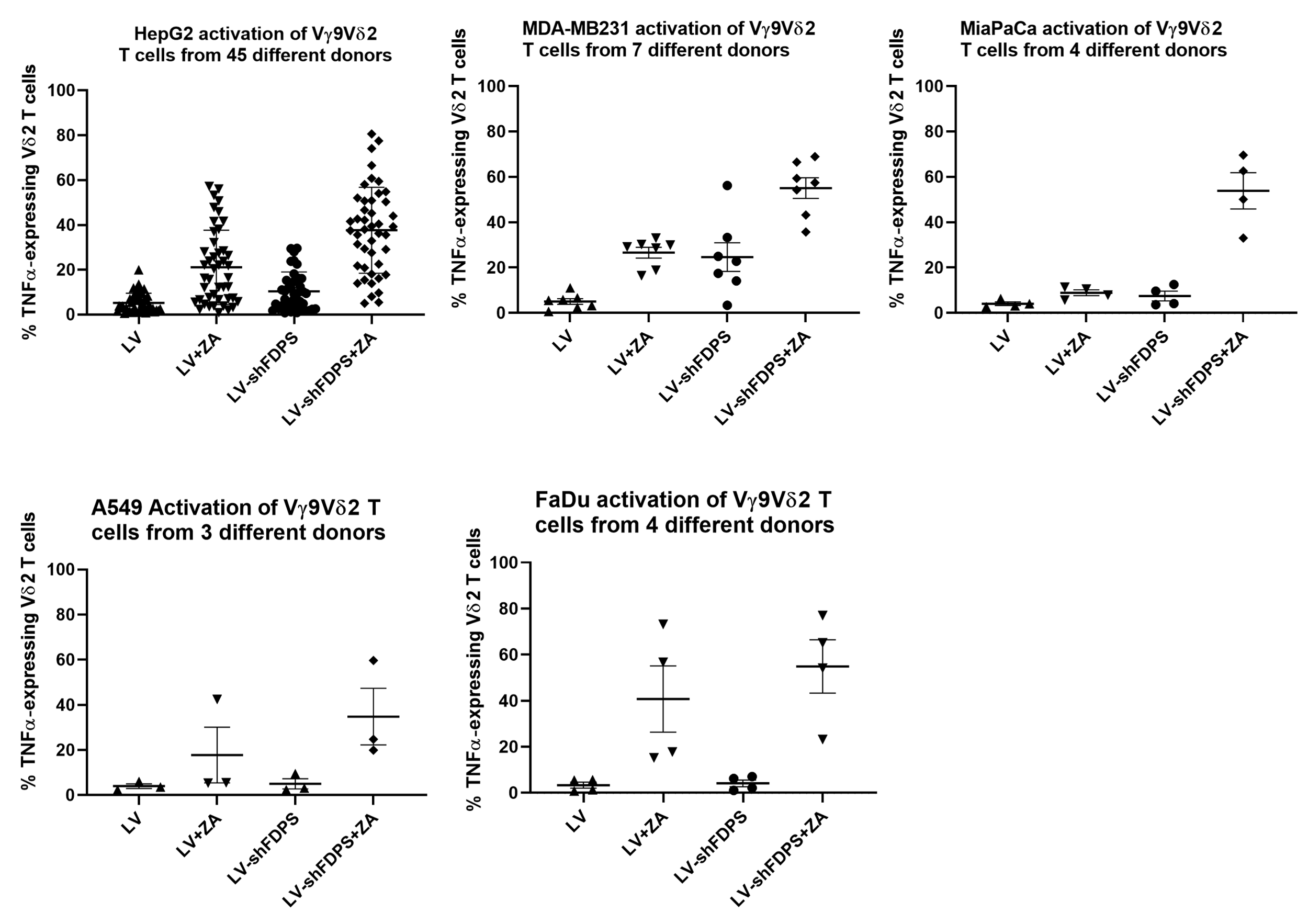
**

**Supplementary Figure 2**. TNFα production is increased in Vδ2 T cells cultured with cancer cells treated with low dose zoledronic acid (ZA) and transduced with shFDPS.

**Supplementary Figure 3.** Tumor-infiltrating Vδ2 T cells were measured by flow cytometry analysis of PC3 tumors transduced with either LV-Control or LV-shFDPS. Mice were injected weekly with 0, 2, 4, or 8 million Vδ2 T cells for 4 weeks. Tumors were extracted and the tumor cells were analyzed by flow cytometry for co-expression of CD45, CD3, and Vδ2. In tumor 1 with a dose of 8 million Vδ2 cells, 7.2% of CD3+ cells were Vδ2 positive. In tumor 2 with a dose of 4 million Vδ2 cells, 45.9% of CD3+ cells were Vδ2 positive and with a dose of 8 million cells, 16.9% of CD3+ cells were positive for Vδ2.


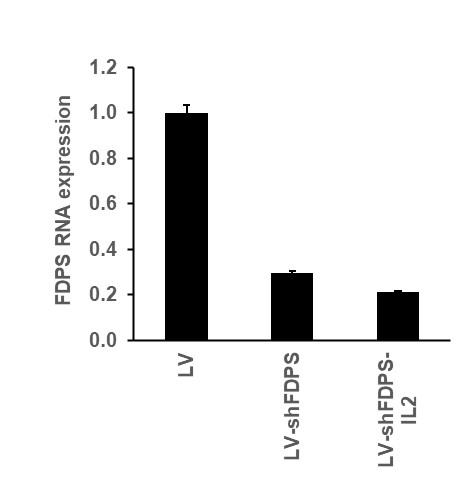


**Supplementary Figure 4**. A lentivirus expressing a short-hairpin RNA (shRNA) targeting farnesyl diphosphate synthase (FDPS) reduces its expression in cancer cells. Real-time qPCR analysis of FDPS RNA from PC3 prostate carcinoma cells transduced with either LV-shFDPS or LV-shFDPS-IL2 at 20 MOI for 72 h. FDPS expression was reduced by 70% with LV-shFDPS and 79% with LV-shFDPS-IL2 as compared with no LV treatment.
